# Grey wolf optimization technique with U-shaped and capsule networks-A novel framework for glaucoma diagnosis

**DOI:** 10.1016/j.mex.2025.103285

**Published:** 2025-03-31

**Authors:** Govindharaj I, Ramesh T, Poongodai A, Senthilkumar K. P, Udayasankaran P, Ravichandran S

**Affiliations:** a Department of Computer Science and Engineering, Vel Tech Rangarajan Dr.Sagunthala R&D Institute of Science and Technology, Tamil Nadu, 600062, India; b Department of Computer Science and Engineering, R.M.K Engineering College, Thiruvallur, Tamil Nadu, 601206, India; c Department of Computer Science and Engineering (Artificial Intelligence), Madanapalle Institute of Technology & Science, Andhra Pradesh, 517325, India; d Department of Artificial Intelligence and Data Science, Kings Engineering College, Chennai, Tamil Nadu, 602117, India; e Department of Artificial Intelligence and Machine Learning, Kings Engineering College, Chennai, Tamil Nadu, 602117, India

**Keywords:** Glaucoma detection, Optic disc segmentation, Grey wolf optimization, U-Net++, Capsule network, Deep learning classification, Vision loss, Grey Wolf Optimized UNet++ with Capsule Network for Automated Glaucoma Screening (GWO-UNet++-CapsNet).

## Abstract

The worldwide prevalence of glaucoma makes it a major reason for blindness thus proper early diagnosis remains essential for preventing major vision deterioration. Current glaucoma screening methods that need expert handling prove to be time-intensive and complicated before yielding appropriate diagnosis and treatment. Our system addresses these difficulties through an automated glaucoma screening platform which combines advanced segmentation methods with classification approaches. A hybrid segmentation method combines Grey Wolf Optimization Algorithm with U-Shaped Networks to obtain precise extraction of the optic disc regions in retinal fundus images. Through GWOA the network achieves optimal segmentation by adopting wolf-inspired behaviors such as circular and jumping movements to identify diverse image textures. The glaucoma classification depends on CapsNet as a deep learning model that provides exceptional image detection to ensure precise diagnosis. The combination of our method delivers 96.01 % segmentation together with classification precision which outstrips traditional approaches while indicating strong capabilities for discovering glaucoma at early stages. This automated diagnosis system elevates clinical accuracy levels through an automated screening method that solves manual process limitations. The detection framework produces better accuracy to improve clinical results in a strong effort to minimize glaucoma-induced blindness worldwide and display its capabilities in real clinical environments.

• Hybrid GWOA-UNet++ for precise optic disc segmentation.
• CapsNet-based classification for robust glaucoma detection.
• Achieved 96.01 % accuracy, surpassing existing methods.

## Graphical abstract

**Figure:**
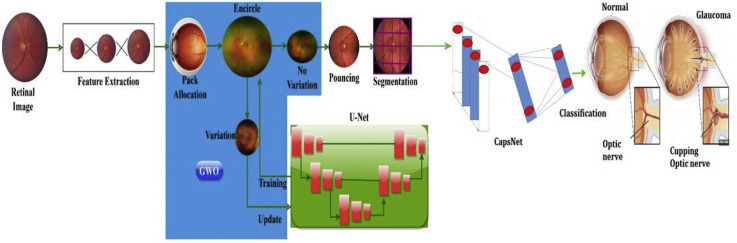

Specifications table

**Table:** 

Subject area:	Computer Science
More specific subject area:	This research focuses on automated glaucoma screening using hybrid optimization-enhanced segmentation and deep learning-based classification techniques. Specifically, it explores the integration of the Grey Wolf
	Optimization Algorithm (GWOA) with UNet++ architecture for precise segmentation of optic disc regions in retinal fundus images. The study then leverages Capsule Network (CapsNet) for robust classification of glaucoma stages, emphasizing improvements in both segmentation accuracy and classification performance for early glaucoma detection.
Name of your method:	Grey Wolf Optimized UNet++ with Capsule Network for Automated Glaucoma Screening (GWO-UNet++-CapsNet).
Name and reference of original method:	1. **UNet++:** Wang, Z., Zheng, J., Zhang, Y., Cui, G., & Li, L. (2024). Mamba-UNET: UNET-Like Pure Visual Mamba for Medical Image segmentation. arXiv (Cornell University). https://doi.org/10.48550/arxiv.2402.05079 2. **Capsule Network (CapsNet):** Afrifa, S., Varadarajan, V., Zhang, T., Appiahene, P., Gyamfi, D., Gyening, R. O. M., Mensah, J., & Berchie, S. O. (2024). Deep learning based capsule networks for breast cancer classification using ultrasound images. Current Cancer Reports, 6, 205–224. https://doi.org/10.25082/ccr.2024.01.002 3. **Grey Wolf Optimization (GWO):** Sengan, S., Lambture, B., Lazar, A. J. P., Benarji, B. S. N., Bakkiyaraj, J., Sharma, D. K., & Lakineni, P. K. (2024). Integrating Grey Wolf Optimizer with U-Net for Precision Retinal Vessel Segmentation in Fundus Images. In Lecture notes in electrical engineering (pp. 377–389). https://doi.org/10.1007/978–981–97–7616–0_27
Resource availability:	**Datasets:** RIM-ONE Dataset (a benchmark dataset for glaucoma detection and optic disc segmentation). https://www.kaggle.com/datasets/ayush02102001/glaucoma-classification-datasets

## Background

The rationale for proposing the GWO-UNet++-CapsNet is formed from the understanding of the pressing urgency of early glaucoma identification and evaluation of its progression [1,2]. It is recognized that glaucoma ranks third on the global list of causes of irreversible blindness, and the main reason for this type of vision loss is damage to the optic nerve. However, patients tend not to show signs of the disease until they suffer a loss of vision in one eye therefore early detection is critical for treatment that can reverse the damage [3,4]. The conventional techniques used in glaucoma diagnosis including clinical examination and visual field tests are skilled based, time-consuming and are often unavailable in resource-limited settings. Therefore, an automated, accurate and efficient diagnostic devise has the potential for positively altering glaucoma care [5].

Our method addresses two crucial challenges in automated glaucoma detection: The features involving

1) Detailed delineation of the open anterior chamber angle and optic disc or cup area in fundus images and
2) Accurate diagnosis and multiclass classification for glaucoma stages prediction.

Most conventional image segmentation methods were designed for images with minimal variation in lighting, contrast, or anatomical differences that might be seen in the human retina. To counter these challenges, we proposed a combination of segmentation and classification where GWOA can boost the performance of UNet++ for segmentation. The GWOA fine tunes the model network configuration to mimic wolf like behaviour such as the encircling and pouncing to extract less easily discernible differences in the textural patterns of the optic disc region, hence creating better segmentations [6].

In the classification phase we used CapsNet people trust this deep learning framework because it achieves great results in identifying images and for its capacity to capture spatial hierarchies of inputs [7]. CapsNet is specifically good at the preservation of spatial information relevant for glaucoma diagnosis because it can maintain important structural relationships within the segmented optic disc and cup regions. When integrating these segmented features, it is thus possible to determine glaucoma stages well and with high detection accuracy besides avoiding high false positive and negative results [8].

This methodology is well suited to the clinical and real-world cases where early, fast and automatic report of the retinal images is shifting to be more imperative. Accordingly, our proposed GWO-UNet++-CapsNet model focuses on the following advantages of automatic glaucoma screening. Finally, it allows glaucoma diagnosis with little manual input. Such an approach could be used as the first-tier assessment of patients in telemedicine settings, rural health clinics, as well as in specialized ophthalmic centres, where clinicians could utilise this tool to identify People who have high risk conditions must be seen before others [9].

However, our method concept could be applied to other ophthalmic diseases where segmentation and classification of optic disc may be necessary, and it can be a boon in medical imaging [10]. The utilisation of this automated framework hence enhances efforts at reducing the disparity in timely diagnosis of glaucoma consistent with the principle of health equity and minimizing the prevalence of blindness caused by glaucoma. In doing so, the present work is situated within the overarching objectives of enhancing patients’ experiences and therapeutic outcomes, optimizing practice activities, and leveraging technology to extend access to high-quality ophthalmological care [11].

**Fig. 1:**
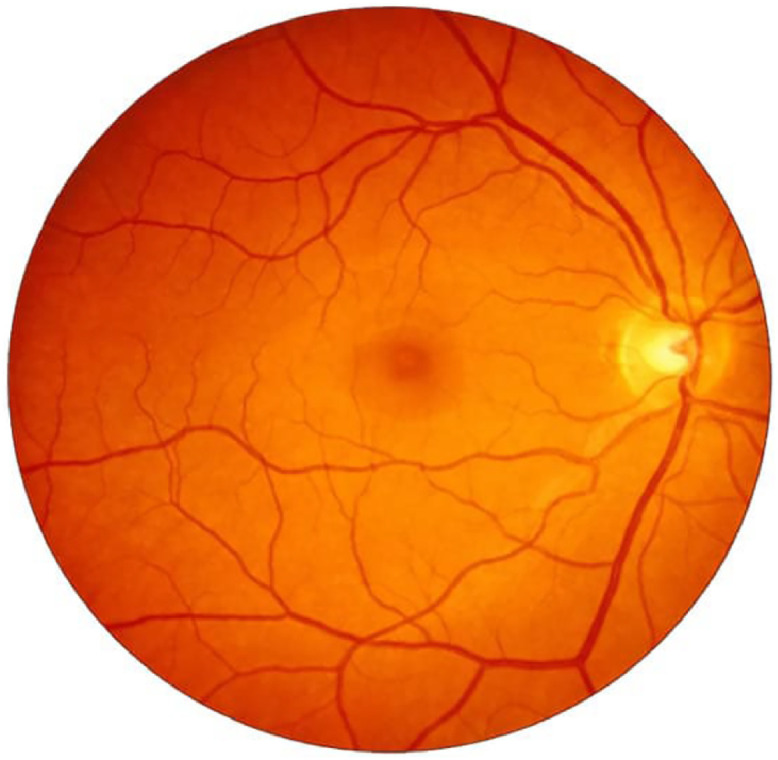
Retinal Fundus Image.

Medical images frequently conceal important signs and necessitate years of specialized expertise for effective interpretation, whereas natural sceneries in Fig. 1 provide features of color along with shape and texture support the detection of disease indicators.

## Method details

Advanced deep-learning approaches have made significant contributions to the detection of glaucoma. To assist patients, maintain their vision by acting early in the course of the disease, DL is employed to detection and track the evolution of glaucoma disease using retinal fundus images.

• To particularly detect optical cups in retinal fundus images, an efficient segmentation technique was created. It provides comprehensive information on retinal fundus images and accurately delineates cup and disc characteristics using UNet++. Inadequate segmentation findings produced by UNet++ have been shown to result in subpar diagnostic quality and, eventually, decreased glaucoma diagnosis efficiency. The Grey Wolf Optimization technique (GWOA), an adaptive optimization technique, is being developed to improve this circumstance. To achieve better segmentation results and, eventually, better glaucoma diagnosis outcomes, this algorithm optimizes UNet++ settings.
• The advent of the CapsNet deep-learning architecture, which enables the diagnosis of glaucoma illnesses, is another noteworthy breakthrough. This innovative approach functions as a sophisticated diagnostic and detection technique. The new glaucoma detection model was thoroughly compared with previously established detection techniques to assess its efficacy and dependability. Its effectiveness and dependability were evaluated using performance criteria.

These findings signal key breakthroughs in the glaucoma screening process and potentially alter early diagnosis and treatment strategies to safeguard vulnerable persons at risk of developing degenerative sickness from losing their eyesight.

This study targets its main research objective to find answers for three specific issues. Our research will center on these research questions which direct our investigation. We want to enhance the quality of glaucoma segmentation and classification methods. Our research explores three methods:

(1) how to improve the accuracy and robustness of glaucoma identification and classification results
(2) how to optimize the segmentation process using Grey Wolf Optimization (GWO) with UNet++,
(3) how to improve, enhance the segmentation and classification in glaucoma detection using Capsules Network.

A deep learning (DL) based glaucoma disease prediction method. Glaucoma detection is a time-consuming task that requires proper data for disease prediction. The developed method uses a cropped optic cup to segment the blood vessels for detection [12]. The DL-based method enhances the feasibility of providing relevant medical and diagnosis services to the users. The developed method maximizes the accuracy of disease prediction. They make extensive use of both machine learning and image processing techniques; for instance, using retinal images as examples, unsupervised learning algorithms help separate optic disc and cup from enhanced retinal pictures, while various machine learning methods to classify pictures into normal or glaucoma categories [13,14].

To eliminate the time-consuming level in [15] introduced a multi-resolution image combination approach with a large kernel residual convolution attention module. The introduced approach analyzes the CDR is used as an important factor for image segmentation. The convolution attention module is employed here to maximize the resolution range of the medical images. The CDR reduces computational cost and latency in the disease detection process. Using a variety of classifiers, including Radial Basis Function classifiers and Multi-layer Perceptron Random Forest classifiers, researchers have recently created new techniques for diagnosing glaucoma [16]. The diagnostic methods used for glaucoma identity consist of heuristics and deep learning technologies. Heuristic methodologies make use of screening techniques that determine the retinal nerve fiber layer (RNFL) thickness and feature extraction through image processing techniques. Additionally, [17] concentrated on enhancing the diagnosis of glaucoma by obtaining higher-order spectral and textural information. In [18,19] Optic Disc (OD) and Optic Cup (OC) are important for fundus image segmentation. This study proposes attention U-Net models with three Convolutional Neural Networks (CNNs) architectures, namely Inception-v3, Visual Geometry Group 19 (VGG19), Residual Neural Network 50 (ResNet50) to segment fundus images. Several data augmentation techniques were used to avoid overfitting and achieve high accuracy.

A research work [20,21] implements three different Convolutional Neural Network (CNN) models including Inception-v3, Visual Geometry Group 19 (VGG19), and Residual Neural Network 50 (ResNet50) for glaucoma patient classification using eye fundus images. Both methods, however, have drawbacks because it is considered a subset of the vision loss images' attributes; hence, their classification accuracy was not very high. The method used features at multiple resolutions to develop joint optic disc and cup segmentation [22,23]. The transition is employed here to analyze the features that are taken from the fundus photos. The transition reduces the computational cost in disc and cup segmentation. The proposed method increases the effective range of fundus images for segmentation. The proposed method enhances the feasibility of the segmentation process [24,25].

A new technique distinguishes it from conventional methods by extracting image features before applying them to a CNN model for conducting normal or abnormal image assessments [26]. A new hybrid graph convolutional neural network (HGCN) [30,31] for the retinal image classification. Combining features from convolutional neural networks into an underlying framework for graph learning based on modularity generates a very powerful classification system uses graph convolution to accurately analyze retinal images based on their characteristic features. Moreover, their research creates a retinal image classifier which uses ensemble-learning as its underlying method [32]. To offer a deep learning approach for retinal image recognition, also exploited U-Net and Inception V3 network designs. Furthermore, developed by integrating U-Net with Efficient Net technology is a model for glaucoma detection in the eyes [33].

The identification of subtle visual characteristics happens autonomously with CNNs because these networks can detect patterns to classify objects based on their distinct features [34]. Medical image segmentation through CNNs is conducted by different U-Net architectures including UNet, 3DUNet, Attention UNet, CeNet, TransUNet, and UNet++. The lack of sufficient training data causes difficulties for these methods that include both overfitting and insufficient learning models [35]. This is frequently the result of inadequate training data, which causes overfitting because of overfitting. When given inadequate data, transfer learning can be a useful strategy [36]. The classification of natural images and more accurate glaucoma detection with fundus images are the main topics of research in this field. Due to their many similar regions, including white backgrounds and the periphery of an eyeball, fundus images provide special challenges in the diagnosis of glaucoma because they may contain irrelevant information that diverts CNN-driven processes [37,38].

Research [43] verifies how convolutional neural networks (CNNs) perform at detecting glaucoma diseases in retinal imagery. The deep learning-based digital extraction techniques reduce the requirement of conducting screening by human operators through manual methods. The research paper [44] describes a CAD system that utilizes fundus photographs to detect early glaucoma through automated analysis. An CNN-based framework within this system enhances diagnostic precision and ensures increased reliability during screenings. Study [45] adopts a mixed technique which unites Region-based CNN (R-CNN) with ResNet-50 along with image segmentation methods. The detection system achieves better accuracy through an approach which joins object detection with enhanced feature extraction while it improves identification between glaucomatous and healthy retinal pictures.

## Methodology

**Fig. 2:**
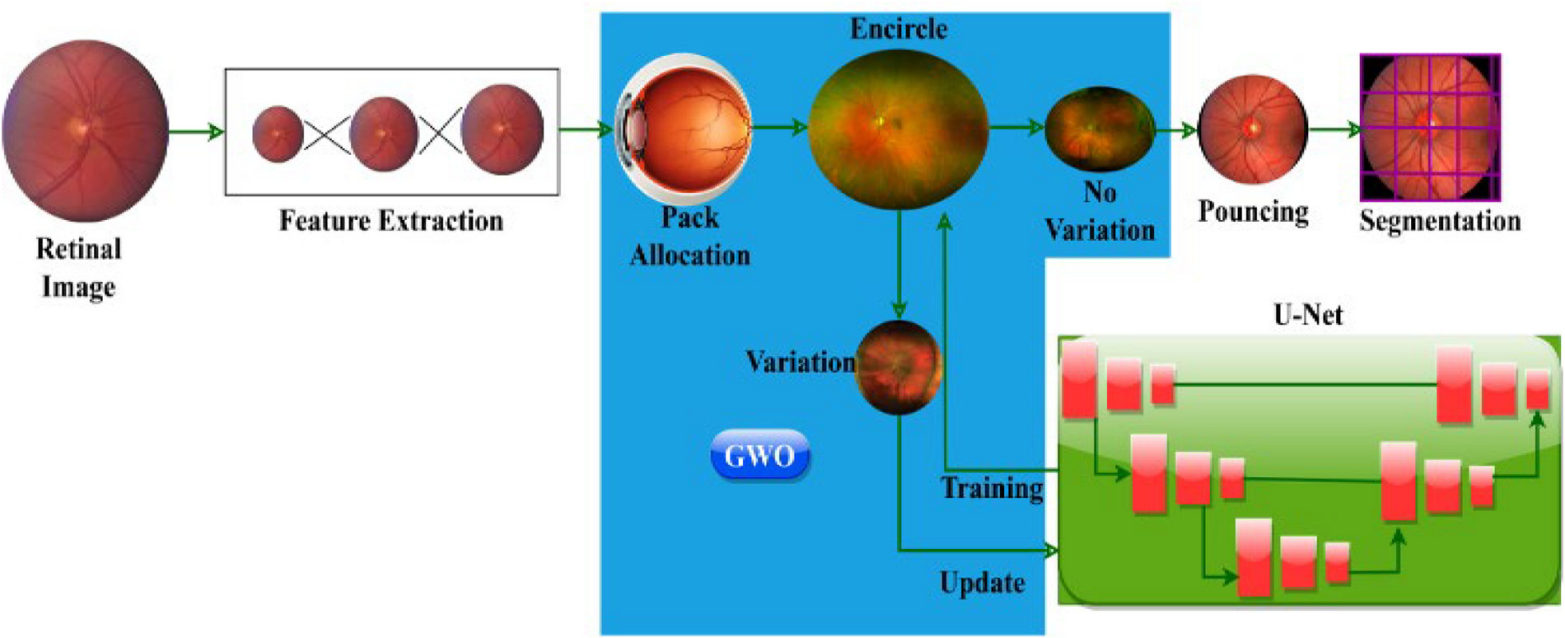
GWO with U-Net Architecture.

In this proposed method, we introduce a hybrid strategy that combines the Capsule Network (CapsNet) and the U-Shaped Network (UNet++) model based on the Grey Wolf Optimization Algorithm. Specifically, the GWOA optimizes the UNet++ parameter in this case, resulting in the exact delineation of optical cup and disc features in retina fundus images/pictures. In the classification phase, the segments characteristics are then used as inputs. Here, we have successfully detected glaucoma using the Capsule Network. The design is to improve Diabetic Retinopathy (DR) recognition accuracy. In this model, a GWO-aided U-Net architecture is implemented for optimal textural feature extraction from input images based on diabetic retinopathy detection. The accurate quality of the retinal image is estimated based on the maximum extraction of the textural feature from which the pack allocation is employed for retaining high-quality features that augment semantic segmentation. Fig. 2 presents the proposed GWO with U-Net Architecture.

For instance, due to diabetes, diabetic retinopathy takes place in retinal blood vessels. The Grey Wolf Optimization is applied to extract all the textural features from input images and then classify them based on their sequence differentiation. Identifying the visible difference in textural features is recognized for accurately segmenting abnormal lesions from input images.

Firstly, a new DR prediction is pursued by using GWO. GWO is applied initially to achieve the most fit circumstances. In this study, the variation causing and No variation features are classified based on pack allocation, encircling, and pouncing measures with GWO. In this model, GWO is used to improve DR recognition accuracy. For accurate DR classification and detection, GWO is utilized with U-Net architecture employed in each dimension to frame semantic segmentation architecture. The traditional U-Net architecture is reframed with adaptable feature maps for identifying the problems that affect the modified structure. Next maximum pouncing features are added with encircling output through training iterations for accurate image segmentation. Lastly, a maximum pouncing textural feature with no variation is taken to compute the accuracy of retinal image segmentation. The U-Net architecture is implemented for precise DR prediction and detection with no textural difference.

### Grey wolf optimization

The Grey Wolf Optimizer (GWO) refers to the grey wolves’ hunting and guidance behavior framework. In this scenario, the initial wolf packs are randomly selected in GWO. The wolves have multiple techniques for hunting based on prey size. Each pack specializes in hunting depending on particular prey species. Using this technique, accurate retinal image segmentation is made to improve DR prediction and recognition. In this model, α,β, andγ represent the first pair, second pair, and third pair for excellent solution. If the preferred targets or other small prey is identified by the wolf, they first allocate the pack to specialized wolves to hunt this target. Secondly, that wolf pack separates and encircles its prey. Thirdly, the wolf packs will chase, pounce, seize, and pull down the preferred target to the ground and then start to eat. Based on the observations, the alpha pair will eat the prey first. An individual grey wolf's position is represented as $\underset{G_{W}}{\to }=\underset{gw_{1}}{\to }+\underset{gw_{2}}{\to }+\cdots +\underset{gw_{k}}{\to }$ in the form of a vector, wherek is the number of wolves.

The search space dimension is symbolized as$\;SS_{D}$.α, β, and γ pairs move near to prey and Ω pair approaches them. Based on the instance, wolf pack hunting is determined as follows:

(i) Pack allocation,
(ii) Encircling prey and
(iii) Pouncing for prey

The optimization part of the above processes is correlated with the detection process as presented below.

**Fig. 3:**
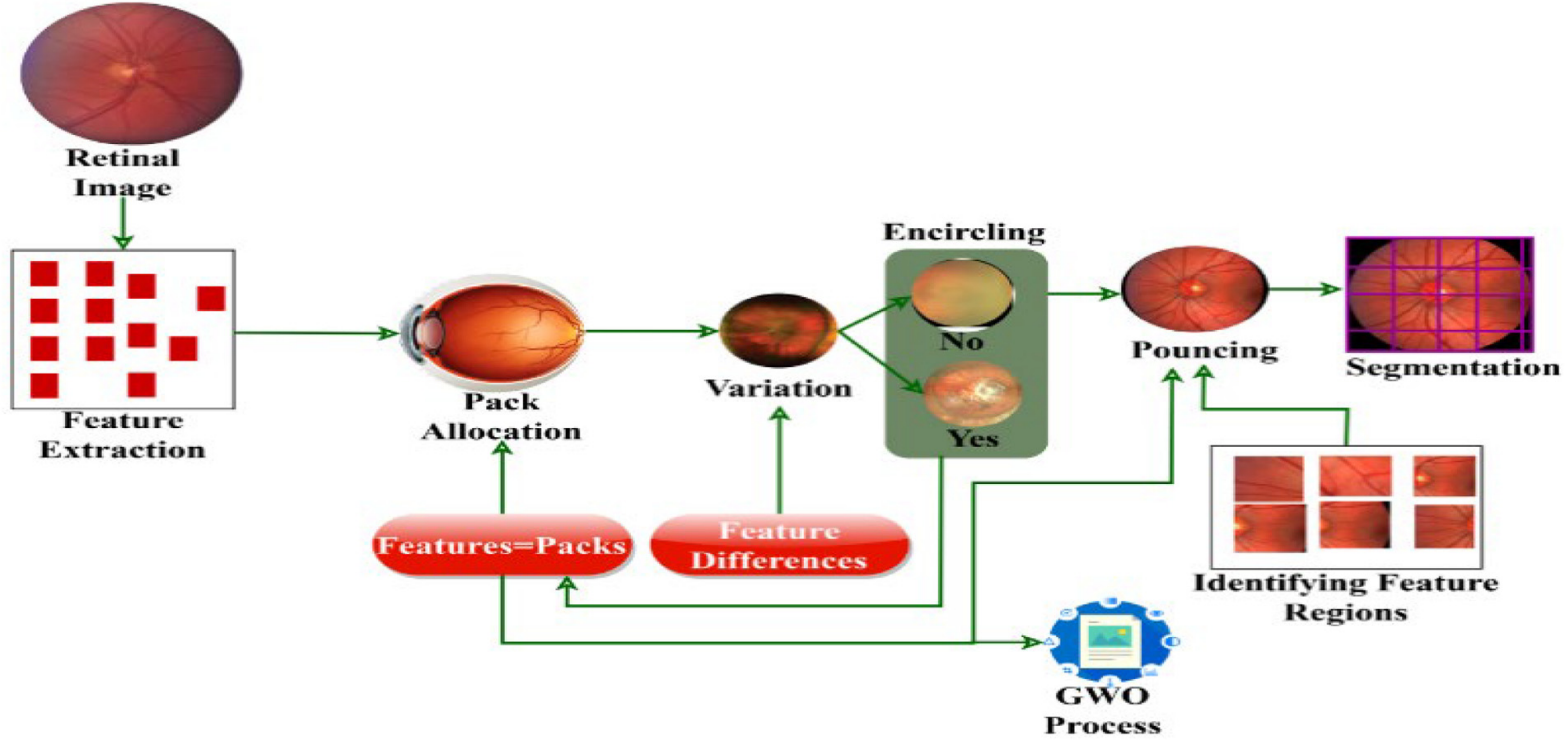
GWO Correlation with the Detection Process.

In the below Fig. 3, the correlation between GWO and the retinal detection process is given. The identified features are allocated for the packs to perform their actions. In this process, the feature differences are identified by the pack behavior and encircling is performed using pouncing activity. These activities/behaviors are used to identify variation feature regions towards segmentation. Here, the pack allocation, encircling (variation), and Pouncing (feature regions) are considered as the GWO Processes.

#### Pack allocation technique

The extracted textural feature served as input resembling the Grey Wolf Optimization. In this model, this requires similar support for encircling and pouncing. Using this technique, the variation-causing and No-variation-causing features are matched using GWO to improve the accuracy of diabetic retinopathy recognition. This textural feature classification depends on different mappings for identifying the maximum encircling and pouncing probabilities during segmentation. Hence, the optimal condition for retinal lesion segmentation from input images is different from the usual technique, which follows the pack allocation by using textural feature classification. This classification is processed for both encircling and pouncing by validating the probability of high and low-resolution features$\;\rho (Rf_{high},\;\;Rf_{low})$ in GWO. The first pack allocation$\;Pack_{a}\left(N\right)$ contains maximum pouncing(P) and sequential textural feature differentiationF(d) is computed as Eq. (1).

(1) $$F\left(d,\;P\right)=\left[ext_{f}-\left(\frac{fc}{fc_{t}}\right)\;\times \frac{1}{\;\rho \left(Rf_{high},Rf_{low}\right)}\right]+Pack_{a}\left(N\right)-1$$

Where, $ext_{f}$, fc and$\;fc_{t}$ are the extracted features from original images, feature classification, and feature classification time for accurate and appropriate pack allocation is defined using GWO. Therefore, the time for adaptable feature maps for segmenting variation occurred lesions is expressed as

(2) $$\sum_{i\in t}=\;\sum_{j\in ext_{f}}Org_{ij}-\sum_{k\in ext_{f}}Ppc_{ij}$$

In Eq. (2), Org and Ppc are the first raw (original) retinal image, and their corresponding pre-processed image is analyzed and compared for accurate pack allocation. The sequential textural feature differentiation ℸF(d,P) depends on P is given as

(3) $$ℸF\left(d,\;P\right)=\sum_{i\in t\;}Pack{\left(N\right)}_{i}-\;\rho {\left(Rf_{high}+Rf_{low}\right)}_{i}$$

In Eq. (3) computes the maximum pouncing relies on sequential textural feature differentiation for $\rho_{Rf_{high}}$ andPack(N). In this scenario, the chances of achieving synchronized modification of featuresω based on α, β, γ, and Ω with the increasing image dimensions from the original image to their corresponding pre-processed image is formulated as

(4) $$\rho_{Rf_{high}}\left(Pack\left(N\right)/fc_{t}\right)=\frac{1}{\sqrt{2ℸF\left(d,\;P\right)\omega^{2}}}experssion\left[1-\frac{ℸF\left(d,\;P\right)-\rho_{Rf_{low}}}{\omega }\right]$$

Where,

(5) $$\omega =F\left(d\right)-Ec_{i}+\rho_{Rf_{low}}*ℸF\left(d\right)$$

**Fig. 4:**
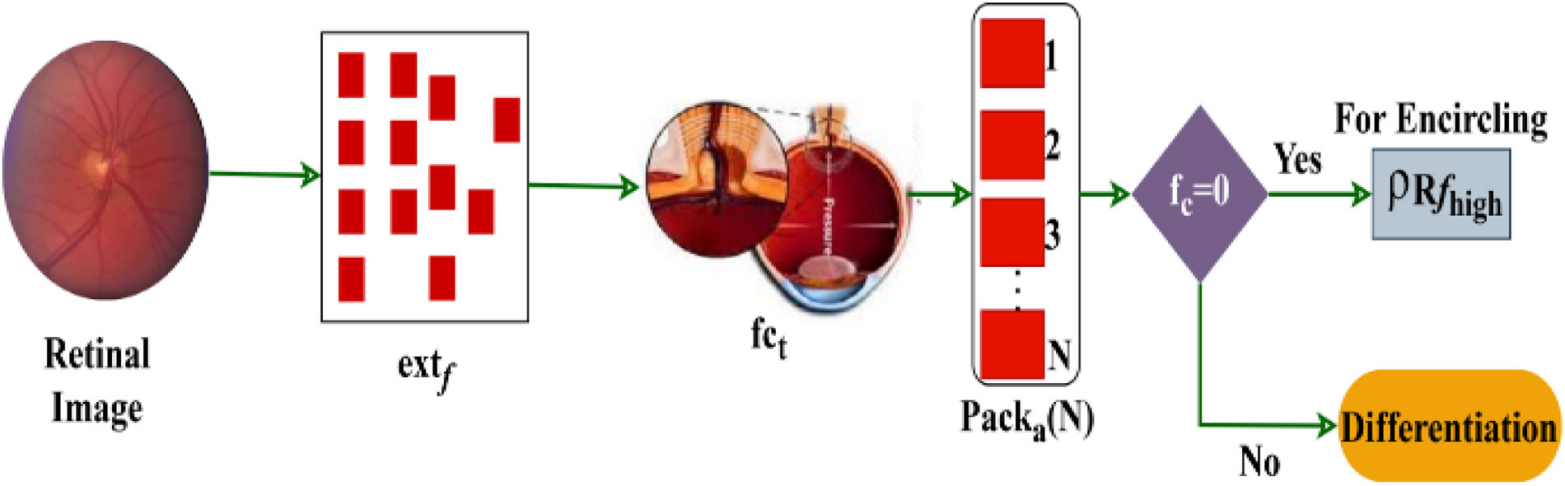
Pack allocation process.

As per Eqs. (4) and (5), the probability assessment is made to reduce the mellitus in input images. This model aims to identify variation causing and No variation features and thereby reduce the textural feature differentiation time for pursuing appropriate pack allocation. The pack allocation process is illustrated in Fig. 4.

The$\;ext_{f}$ is identified from the input image for different textural patterns for classification. In the $\;fc_{t}$ process, $\;Pack_{a}\left(N\right)$ is assigned until $\;ext_{f}$ is true. Based on the variation offc=0 then$\;\rho Rf_{high}$ is the input for encircling and the failing case results in feature differentiation. This process is referred to as pack allocation between different features extracted in Fig. 4.

#### Encircling prey technique

In this technique, the wolf pack changes their positions at the time of optimization for theα,β, andγ pairs based on prey locations. Hence, it is expressed as

(6) $$\underset{E}{\to }=\underset{x}{\to }.\underset{p_{L}\left(t\right)}{\to }-\underset{G_{W}\left(t\right)}{\to }$$

Where,

(7) $$G_{W}\left(T+1\right)=\underset{p_{L}\left(t\right)}{\to }-\left(\underset{x}{\to }\underset{y}{\to }\right)$$

From the above equation, T is the current training iteration, $\underset{p_{L}}{\to }$ and$\;\underset{G_{W}}{\to }$ represents the prey location and the wolf pack position. Where,$\;\underset{x}{\to }$ and$\;\underset{y}{\to }$ signifies the coefficient vectors. For instance, diabetes mellitus $\underset{m}{\to }$ is linearly reduced throughout the training iteration. This computation is pursued to approach the outcome range. In this assessment, the variables$\;\underset{v_{1}}{\to }$ and $\underset{v_{2}}{\to }$ is the random vector between the 0 and 1 range, respectively.

(8a) $$\underset{x}{\to }=\underset{2m}{\to }.\left(\underset{v_{1}}{\to }+\underset{v_{2}}{\to }\right)-m$$

(8b) $$\underset{y}{\to }=\underset{2v_{2}}{\to }$$

And,

(8c) $$\underset{m}{\to }=\frac{1}{2}\left(1-\frac{\underset{y}{\to }}{T}\right)$$

**Fig. 5:**
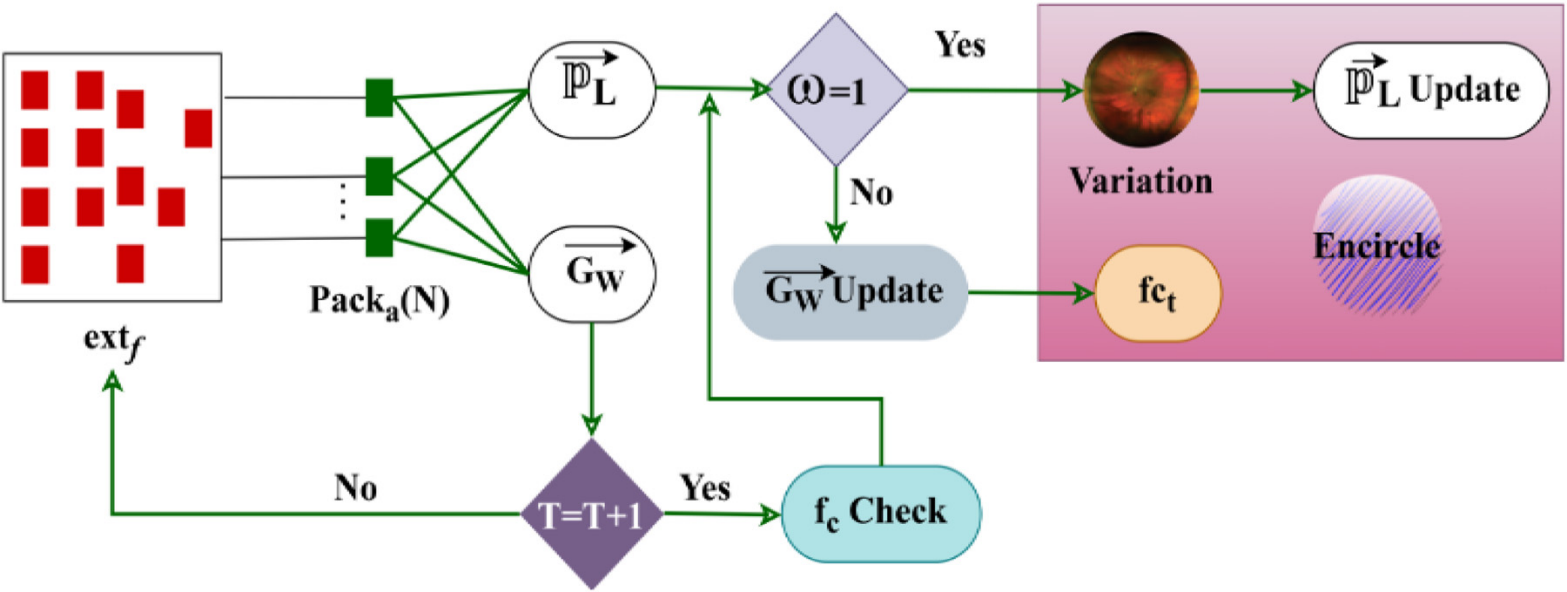
Variation differentiation for encircling process.

Based on the above Eq. (8c), the encircling process for variation is performed. In this case, the variation detection follows $\;\rho Rf_{high}$ or low feature classification. Such classification relies on the maximum feature extraction or fc=0 cases. This variation differentiation for encircling is illustrated in Fig. 5.

The above Fig. 5 presents the encircling process for $\;Pack_{a}\left(N\right)$ through $\;\vec{P_{L}}$ and $\vec{G_{N}}$ updates. This considers ω changes and training improvements between different intervals of variation. The variation is likely to occur between dusting extraction untilω=0. If this case fails, based on the differential features,$\;fc_{t}$ and $\;\vec{P_{L}}$ update performs the encircling process. Therefore, from the $\;Pack_{a}\left(N\right)$, the ω=1 features are extracted until a new variation is detected. This classifies the variation and no-variation instances across different$\;ext_{f}$.

#### Pouncing for prey

Pouncing behavior is performed by the three pairs ofα,β, andγ wolf pairs. Because they are superior and specialized in identifying prey locations thereby reducing additional searching time. The three great outcomes are used by Ω pairs for updating their location.

(9a) $$\underset{E_{\alpha }}{\to }=\left|\underset{y_{1}}{\to }.\underset{G_{W_{\alpha }}}{\to }-\underset{p_{L}}{\to }\right|$$

(9b) $$\underset{E_{\beta }}{\to }=\left|\underset{y_{2}}{\to }.\underset{G_{W_{\beta }}}{\to }-\underset{p_{L}}{\to }\right|$$

(9c) $$\underset{E_{\gamma }}{\to }=\left|\underset{y_{3}}{\to }.\underset{G_{W_{\gamma }}}{\to }-\underset{p_{L}}{\to }\right|$$

Where, $\underset{E_{\alpha }}{\to }$ and $\underset{G_{W_{\alpha }}}{\to }$ symbolize the current and modified alpha pair location whereas $\underset{E_{\beta }}{\to }$ and $\underset{G_{W_{\beta }}}{\to }$ shows the current and updated beta pair location. Lastly, $\underset{E_{\gamma }}{\to }$ and $\underset{G_{W_{\gamma }}}{\to }$ signifies the current and updated gamma pair location. Post distance measurement, the accurate location of the current pair$\;\underset{G_{W_{1}}}{\to }$, $\underset{G_{W_{2}}}{\to }$ and $\underset{G_{W_{3}}}{\to }$ is formulated as

(10) $$\underset{G_{W_{1}}}{\to }=\underset{G_{W_{\alpha }}}{\to }-\underset{x_{1}}{\to }.\underset{E_{\alpha }}{\to },\;\;\underset{G_{W_{2}}}{\to }=\underset{G_{W_{\beta }}}{\to }-\underset{x_{2}}{\to }.\underset{E_{\beta }}{\to },\;\;\underset{G_{W_{3}}}{\to }=\underset{G_{W_{\gamma }}}{\to }-\underset{x_{3}}{\to }.\underset{E_{\gamma }}{\to }$$

In this model, the encircling prey is referred to as exploration whereas pouncing prey is referred to as exploitation; the grey wolf pack varies from each other; they converge.

### Textural difference identification

**Fig. 6:**
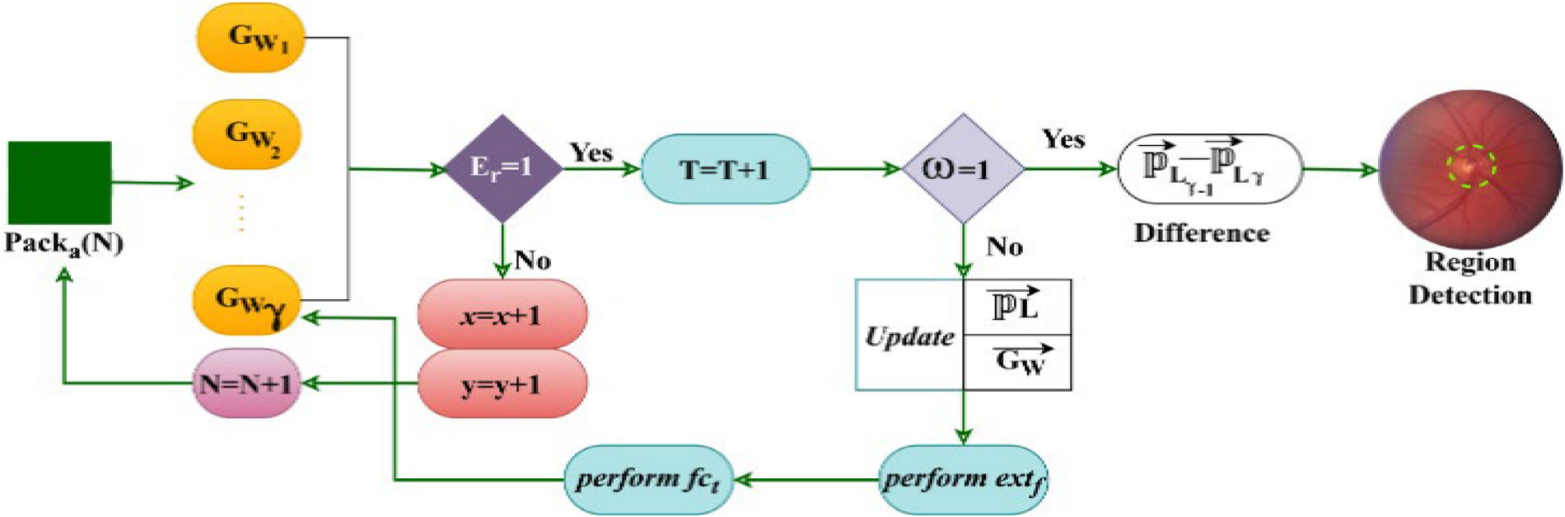
Textural Difference Identification.

Early prediction and recognition of DR are performed to provide precise diagnosis. This process can reduce the chances of the issue in eye structure. The feature differentiation time for all the allocated packs is computed without variation. In this study, the present state-of-the-art model used for diabetic retinopathy prediction based on semantic segmentation is performed in input retina images. In this scenario, the sequential differentiation from extracted features is handled, where training and updating are balanced through U-Net architecture to improve recognition accuracy. Hence, the segmentation range determines the textural feature extraction and differentiation along with computation time. The U-Net architecture classifies variation, and No variation features based on providing training to the original image. The textural difference identification process is illustrated in Fig. 6.

The $\;Pack_{a}\left(N\right)$ decides the textual difference detection from its movement and position. First, if$\;E_{\gamma }=1$ (True) then training iterate is incremented to check if variations are present. If this is true then the region is identified as the difference between $\;\vec{P_{L}}$ vectors across different locations. This region is segmented for further DR infection detection. The ω=0 generates no variation from which $\;\vec{P_{L}}$ and $\;\vec{G_{N}}$ updates are pursued to extract new features. Considerably the $\;E_{\gamma }=0$ update $\;\vec{x}$ and $\;\vec{y}$ for increasing the chances of$\;\;GW_{\gamma }$ or the pack count. The higher the pack count; the higher the segmenting ω=1/detection rate (Fig. 5). After image pre-processing, the actual goal of this model is to identify the retina quality using fundus images. The fundus images are processed through the U-NET architecture for performing the optimal image segmentation. In this architecture, the training images and segmented images are compared to identifying the variation causing and No causing features from the input alone.

### Training U-Net architecture

U-Net architecture is implemented to increase the maximum pooling layers and textural feature differentiation based on accurate image segmentation is performed by using exploration and exploitation way. This architecture is applied to perform the cross-connections between exploration and exploitation to achieve great outcomes. The exploration method of U-Net includes four units namely Encoding units, Decoding units, Up-sampling units, and Down-sampling units. The individual encoding unit consists of two types of pack allocation with maximum variation-causing features. In each pooling layer, the adaptable features maps are packed with GWO. The center region between exploration and exploitation way is otherwise known as a bottleneck. In this model, this bottleneck has two types of pack allocation (i.e.) high-resolution features (Up-sampling) and low-resolution features (Down-sampling). From the instance, in the decoder unit, four decoders are holding to frame the exploitation way by using the U-Net architecture.

**Fig. 7:**
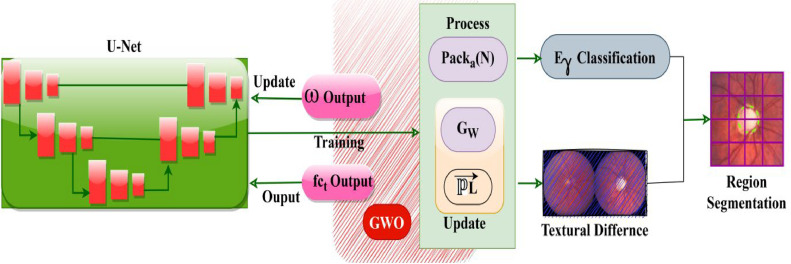
U-Net Architecture for Proposed GWO Process.

This architecture is implemented for accurate retinal image segmentation in fundus photography to improve the accuracy of DR recognition. This U-Net performs DR prediction and recognition with corresponding pre-processed retina images from which the textural variation causing features are reduced. The U-Net architecture is implemented in this article to identify and segregate the maximum pouncing and variation-causing features from the given datasets for precise retinal image segmentation. A textural feature differentiation is performed to detect the key factors in input retina images and thereby reduce the risk of diabetic mellitus during feature extraction. The U-Net architecture for the proposed GWO-based optimization is illustrated in Fig. 7.

The U-Net architecture relies on ω and $\;fc_{t}$ outputs for initiating$\;Pack_{a}\left(N\right)$ and $\left(\;\vec{G_{W}}\;\vec{P_{L}}\right)$ updates. In this process $\;E_{\gamma }$ classification and $\;P_{L}$ differences are used to identify region segments. Based on the U-Net training process the $\;fc_{t}$ and ω variations are used to initiate new$\;Pack_{a}\left(N\right)$ until the T=T+1 is initiated. This process is initiated to ensure $\;\vec{G_{W}}$ and $\vec{P_{L}}$ are updated periodically for $\;E_{\gamma }$ classification and textural difference suppression. In the encoder unit, additional pooling layers are added to reduce the impact. Hence, this architecture identifies low-resolution features that are mapped by understanding high spatial information after pack allocation. This U-Net obtains additional parameters and weights to retain high-resolution features from pre-processed images that improve semantic segmentation and frame the network difficult to train. Firstly, the segmentation architecture is constructed by modified traditional U-Net architecture with adaptable feature maps for accurately segmenting abnormal lesions from the input retina images.

The usual U-Net architecture is a frame to identify the region of interest (ROI) in input retinal images for textural feature differentiation in both the original and pre-processed images. This U-NET contains less data with high prediction and recognition accuracy. Even though the varying weights and parameters are randomly initialized in the updated U-Net, it leads to an over-fitting issue that impacts the segmentation process. The model's accuracy and precision could be enhanced by modifying the traditional way of Up-sampling operations with various sampling for retaining the original textural features after image pre-processing. In this model, this condition fails to maintain linear textural features in images and gives a blurred image. While retina image processing using GWO, the pixel rate varies based on variation causing and No variation causing feature occurrence in the output image is constant.

Pack allocation uses the feature differences as weights where its assessment obtains in each dimension only one addition, thereby enhancing the addition of the pooling layer by using U-Net architecture. The second process is to perform image segmentation using the ReLU activation function for identifying maximum pouncing by again framing encoder and decoder networks respectively. The GWO utilized with U-NET architecture reduces the computational cost of DR prediction and recognition. The U-NET architecture is implemented to reduce the textural variation identification time and thereby increase the precision. The proposed architecture uses adaptable feature maps to enhance the pooling layer-wise networks.

Using U-Net training architecture enables the system to learn various elements from original images while preserving low error percentage. The proposed design achieves better precision and accuracy in detecting diabetic retinopathy through semantic segmentation as well as financial cost reductions. This approach enhances the reliability of retinal image segmentation process. The U-Net framework reaches high accuracy levels for DR detection when combined with dense layers along with down-sampling and up-sampling components. The precise segmentation between textural features of ReLU activation function results in binary-formatted outputs used for accurate image segmentation.

**Fig. 8:**
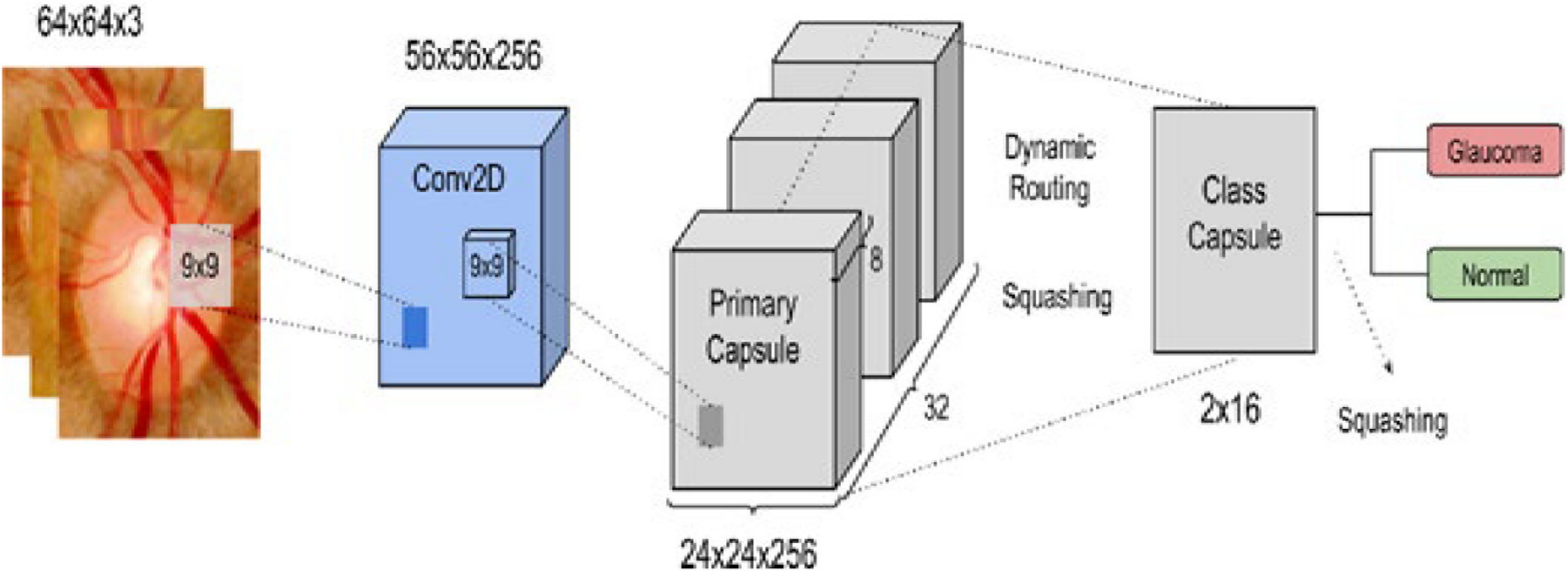
Caps-Network for reliable identification of Glaucomat or Normal.

In Fig. 8 represents a deep learning architecture specifically designed for the classification of retinal images to detect glaucoma.

The model processes the retinal image through multiple layers, extracting features and learning the spatial relationships that are crucial for identifying glaucoma. Capsule networks are particularly effective for medical image analysis because they maintain spatial hierarchies and can more accurately detect features that may indicate the presence of glaucoma, such as changes in the optic nerve head or the retinal nerve fiber layer. This architecture leverages the strengths of capsule networks, particularly their ability to recognize patterns in images, even when the patterns are spatially transformed, making them well-suited for medical image classification tasks like glaucoma detection. The model takes as input a retinal image of size 64 × 64 pixels with three color channels (likely RGB). This input image is a cropped or resized version of a retinal scan, which the model will analyze to determine if the eye shows signs of glaucoma.

The first layer in the model is a 2D convolutional layer that uses a 9 × 9 filter (kernel) to scan across the input image. This layer extracts low-level features such as edges, textures, and gradients. The output of this layer is a feature map of size 56 × 56 × 256, where: 56 × 56 is the spatial dimension, reduced due to the convolution process, 256 is the number of filters applied, each producing a feature map. To capture important features from the retinal image that might be indicative of glaucoma, such as the shape and texture of the optic nerve. The feature map from the Conv2D layer is passed through the Primary Capsule Layer, which groups the feature maps into small vectors (capsules) and applies a squashing function. This function ensures that the output vectors have a magnitude between 0 and 1. The result is a set of 32 capsules, each containing an 8-dimensional vector, arranged in a 24 × 24 grid, resulting in a 24 × 24 × 256 output. To capture spatial hierarchies and relationships in the retinal image, such as the orientation and position of the optic nerve relative to other structures.

The dynamic routing algorithm determines how strongly each capsule in the Primary Capsule Layer should influence the capsules in the next layer (Class Capsule Layer). Dynamic routing iteratively adjusts these connections based on the current input. The Capsule layer contains two capsules, each representing one of the possible classes: “Glaucoma” and “Normal.” Each capsule is a 16-dimensional vector. The squashing function is applied again to produce output vectors whose length corresponds to the probability of the image belonging to each class. The output is magnitude of the vectors in these capsules is used to make the final classification. The network outputs two values corresponding to the likelihood of the input image being classified as either Glaucoma or Normal.

### Method validation

The implementation details regarding the libraries used and the hardware specifications for our research work.

#### Implementation details

(i) Programming Language & Frameworks:
  • The model was implemented using Python 3.8.
  • Deep learning frameworks: TensorFlow 2.8 and PyTorch 1.10 were used for training and evaluation.
  • Image processing: OpenCV 4.5 and PIL (Pillow) were used for preprocessing and augmentation.
  • Optimization algorithms: The Grey Wolf Optimization (GWO) algorithm was implemented using the Scikit-Optimize library.
  • Scientific computing: NumPy and SciPy were used for matrix operations and statistical analysis.
(ii) Hardware Specifications:
  • GPU: NVIDIA RTX 3090 (24GB VRAM) was used for accelerated training and inference.
  • CPU: Intel Core i9–12,900 K (16 cores, 24 threads) for data preprocessing and model evaluation.
  • RAM: 64GB DDR5 for handling large image datasets efficiently.
  • Storage: 2 TB NVMe SSD for fast data access and model checkpoints.
(iii) Training Details:
  • The model was trained using Adam optimizer with a learning rate of 0.0001.
  • Batch size: 32 for effective GPU memory utilization.
  • Epochs: 100, with early stopping to prevent overfitting.

**Fig. 9:**
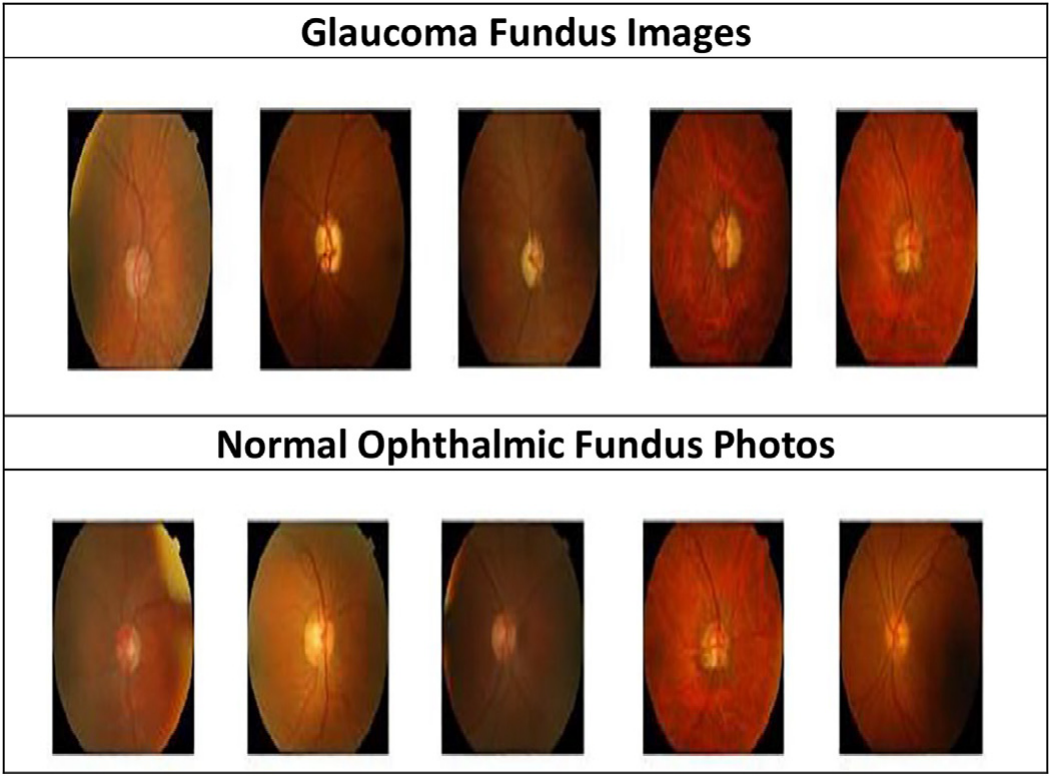
A Case Image from the ORIGA Fundus DataSet.

Our glaucoma detection and categorization technique received testing through several evaluation measures. The research team performed experiments based on the publicly available ORIGA database within the MATLAB environment while maintaining consistent dimensions throughout the tests. The ORIGA dataset contains 650 examples that include 168 pictures of glaucoma-affected eye regions together with 482 images of healthy eyes. However, several abnormalities within the dataset pose significant challenges for classification. Studies evaluate the size along with color variations of optic discs (OD) and optic cups (OC), besides noise-derived blurring that affects intensity and color representations as visualized in Fig. 9. In Fig. 9, these aberrations are also discernible by picture enhancement techniques that give correct portrayal of eye conditions for both classification tasks.

Our dataset was split in an 80:20 ratio to make training and testing easier. This ensures that 80 % of data will be used to train the model, with the remaining 20 % being set aside to evaluate its performance. The development of automated algorithms to identify and categorize glaucoma-affected regions hinges on the proper identification of lesions on optical disc heads [39,40]. We experimented with the ORIGA dataset as our assessment foundation to examine the localization capabilities of our Grey Wolf-based U-Net++ model.

**Fig. 10:**
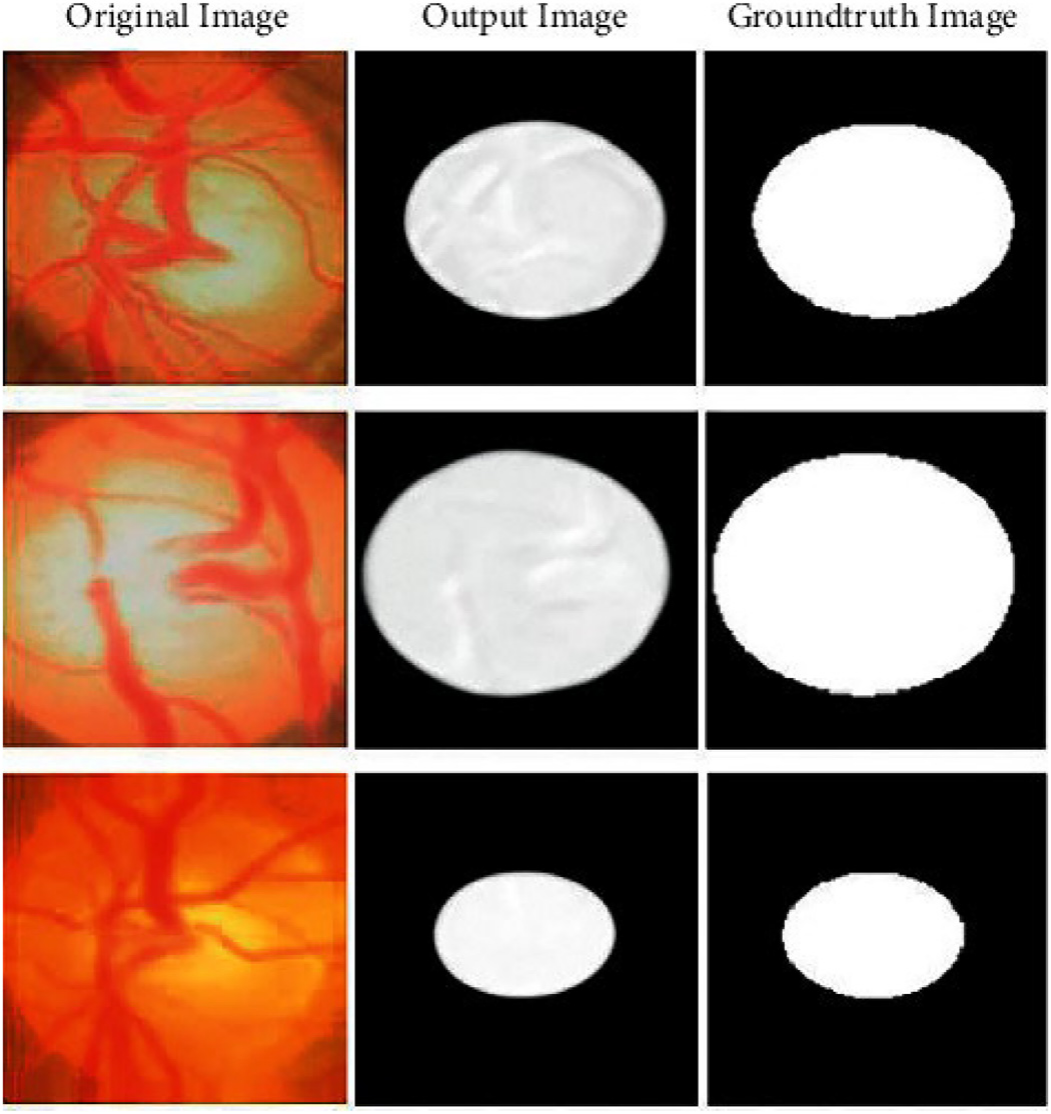
Segmented output.

The outcomes of our studies adopting this strategy are displayed in Fig 10. Regardless of size or location, our recommended Grey Wolf-based U-Net++ model identifies lesions in the optic disc (OD) and cup (OC) with exceptional accuracy. Furthermore, this model is successful for identifying and categorizing glaucoma due to its robustness in managing frequent sample aberrations like blurring, color fluctuations, and brightness variations; Fig. 10 displays the segmented output from the U-Net++ model. Following the Glaucoma, segmentation of the optic disc and cup is effectively classified using the Capsule Network (CapsNet). To successfully classify glaucoma, the segmented output is loaded into the Capsule Network (CapsNet). We carried out a full comparative analysis utilizing cutting-edge approaches on the same dataset as evaluation to demonstrate the efficiency of glaucoma's classification models. We routinely compared the findings of our technique with those reported in references [22,31,32,[33], [34], [35]] to ensure objectivity throughout our review process.

Table 1, shows how the suggested Model GWO with (UNet++) and MRSNet compare in terms of performance.

**Table 1:** Accuracy in (U-Net++) + CapsNet VS Grey wolf based UNet++ with CapsNet.

Models	Accuracy in ( %)
MRSNet [22]	95.8
Grey wolf based UNet++ with CapsNet [Proposed]	96.01

**Fig. 11:**
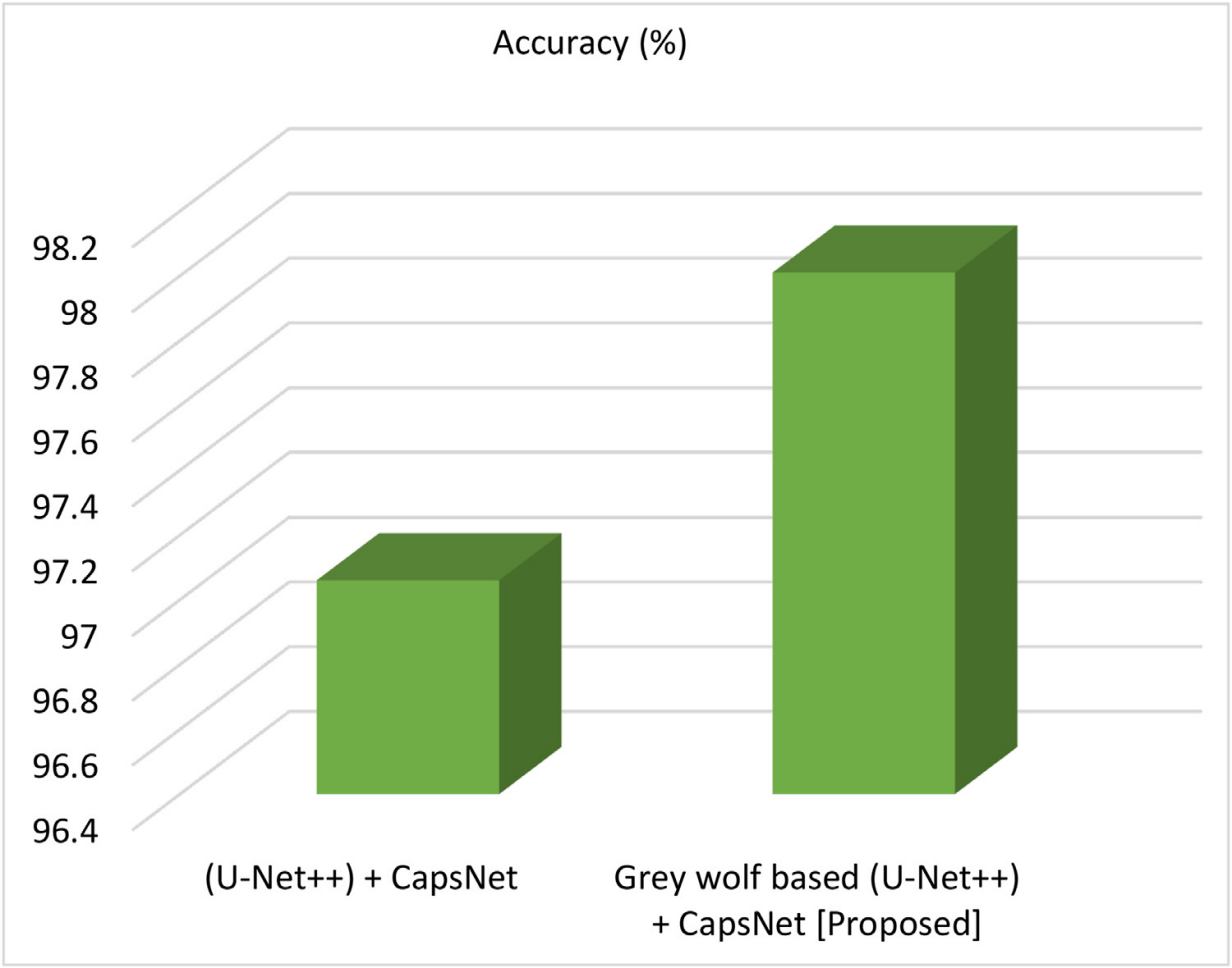
Accuracy testing of the suggested Vs. (UNet++) + CapsNet.

In comparison, our suggested grey wolf based (U-Net++) + CapsNet gives more trustworthy findings across all evaluation metrics. In specifically, it achieves 96.01 % accuracy's, which is significantly greater than previous work. Additionally, Fig. 11 offered the same contrast.

**Fig. 12:**
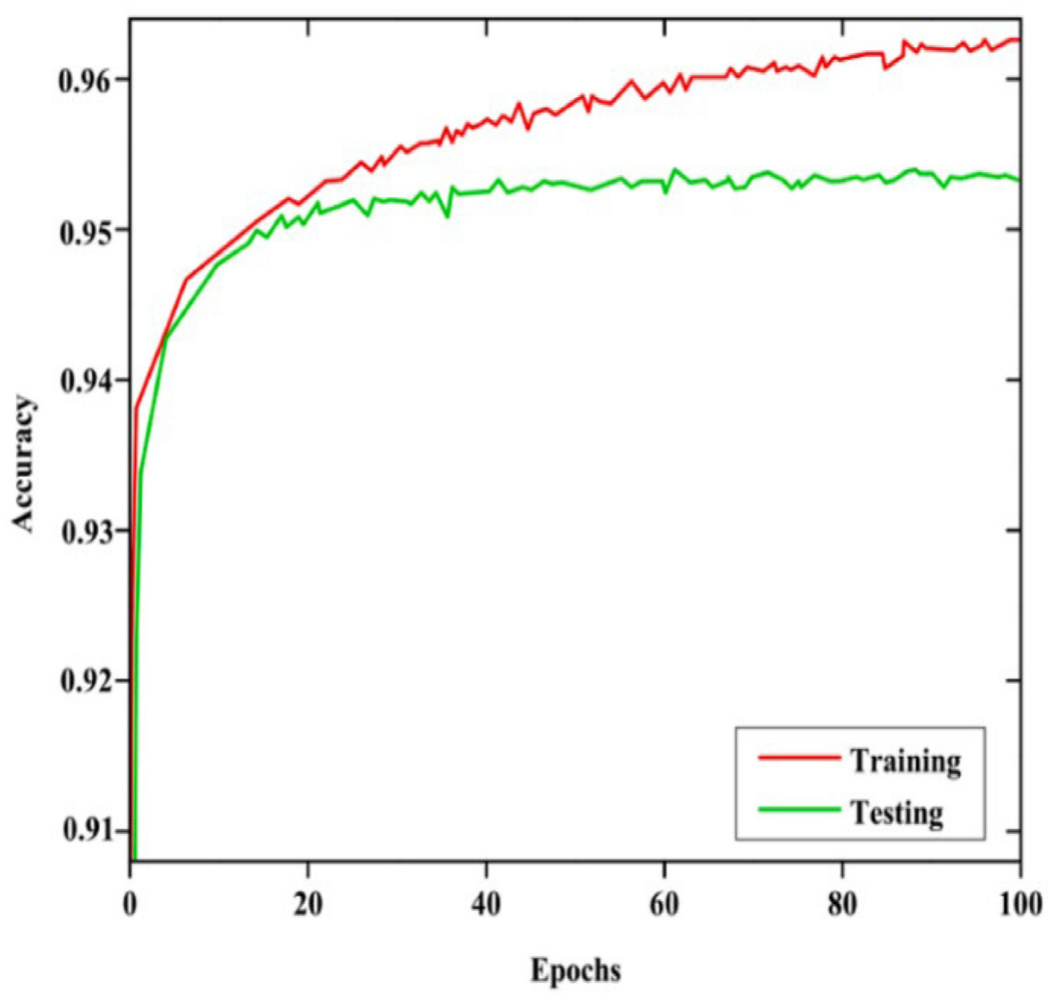
Accuracy Metrics for Training and Testing.

**Fig. 13:**
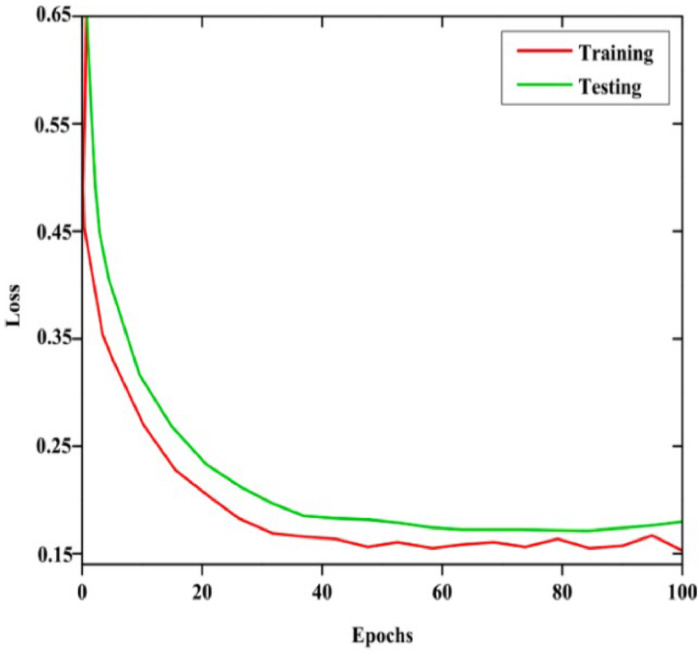
Loss Metris for Training and Testing.

Both Grey Wolf-based U-Net+ and CapsNet operate after the segmentation of optic cup and optic disc to achieve efficient glaucoma classification. The model performance metrics are displayed through Fig. 12 for accuracy and Fig. 13 for loss. The segmented output functions as input to CapsNet for achieving precise glaucoma classification. The optical analysis of model classification appears in Fig. 12 followed by Fig. 13.

Quantitative metrics are presented in Table 2 to help assess efficacy and superiority over alternative treatments. The validation of our proposed GWO-UNet++ and CapsNet-based framework, further reinforcing its effectiveness in glaucoma detection.

**Table 2:** Comparison of the Suggested Method with the Proposed method.

Method	Accuracy ( %)	Precision ( %)	Recall ( %)	Specificity %)	Sensitivity ( %)
MTA-CS [37]	91.5	91.3	92.3	91.4	91.3
ResFPN—Net [27]	93.9	92.0	92.2	91.4	90.7
AGCT [28]	92.6	93.7	92.9	92.5	91.3
E-Net [29]	92.7	93.8	94.2	94.1	93.8
DAGCN [36]	94.8	94.1	93.8	93.7	94.1
MRSNet [22]	95.8	94.2	94.3	94.8	95.4
Proposed (GWO + UNet)	96.01	97.12	97.31	97.34	97.56

Our method achieves 96.01 % segmentation accuracy and 96.01 % classification accuracy, surpassing conventional CNN-based and region-based approaches. The integration of Grey Wolf Optimization (GWOA) with UNet++ enhances optic disc segmentation, ensuring improved feature extraction. Table 3, which presents a comparative analysis of our proposed GWOA-UNet++ & CapsNet framework against state-of-the-art glaucoma detection methods.

**Table 3:** Comparison of Glaucoma Detection Methods.

Method	Segmentation Accuracy ( %)	Classification Accuracy ( %)	Key Features
LCD-CT-CNN (2023)	93.12	94.50	CNN-based classification with traditional segmentation.
HSC-LCD-CT (2023)	94.30	95.20	Hybrid segmentation with CNN-based classification.
ALC-DNN (2024)	95.00	95.80	Deep learning with region-based segmentation.
**Proposed GWOA-UNet++ & CapsNet**	**96.01**	**96.01**	Hybrid Grey Wolf Optimization (GWOA) with UNet++ and CapsNet-based classification.

The proposed model in Table 4 reaches a classification accuracy of 96.01 % to become the most accurate compared to references [17,18,41,42]. The model proves successful in diagnosing glaucoma through its capability in segmentation tasks and classification tasks. By integrating GWO-enhanced UNet++ for segmentation and CapsNet for classification. The comparison evaluates segmentation techniques, classification approaches, datasets, and accuracy level. Our proposed approach integrates Grey Wolf Optimization (GWO) with UNet++ for enhanced optic disc and optic cup segmentation, combined with Capsule Networks (CapsNet) for robust classification. This framework outperforms previous U-Net and CNN-based methods, achieving a higher accuracy of 96.01 % compared to existing models. This demonstrates significant advancements in automated glaucoma screening.

**Table 4:** Comparison of performance metrics and its key features.

Study	Method1	Method2	Dataset	Accuracy ( %)	Contributions
[17]	Attention U-Net	Segmentation	REFUGE, RIM-ONE	95.10	Utilizes Attention U-Net for improved segmentation of the optic disc and cup.
[18]	Classification	CNN	ACRIMA, REFUGE	94.80	CNN-based classification of fundus images for glaucoma detection.
[41]	U-Net	CNN	RIM-ONE, DRISHTI-GS	94.20	Uses U-Net for segmentation and CNN for classification, achieving moderate accuracy.
[42]	Explainable Segmentation & Classification Framework	Deep Learning-based Classifier	RIM-ONE, REFUGE, ACRIMA	95.50	Introduces an automated glaucoma detection system with explainability.
Proposed Method	GWO—Optimized UNet++	Capsule Network (CapsNet)	ORIGA	96.01	Grey Wolf Optimization (GWO) enhances segmentation; CapsNet improves feature extraction and classification robustness.

The Key novel contributions of our approach include:

• GWO—Optimized UNet++: Unlike traditional U-Net-based methods, our approach dynamically optimizes segmentation parameters for improved optic disc and cup extraction.
• CapsNet for Classification: Unlike CNN-based classifiers, CapsNet preserves spatial relationships in images, leading to higher robustness in detecting glaucoma.
• Higher Performance: Our approach achieves higher accuracy than previous studies, demonstrating its superiority in automated glaucoma detection.

**Fig. 14:**
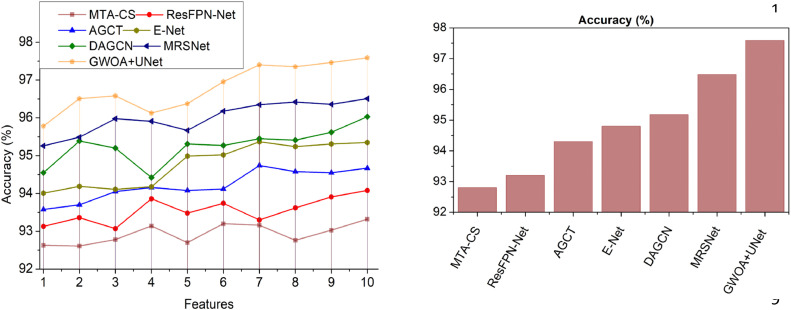
Accuracy.

This section utilizes defined metrics for a comparative analysis, considering two key variants: features (Fig. 14–Fig. 18) and methods. The comparison includes MTA-CS, ResFPN—Net, AGCT, E-Net, DAGCN, and MRSNet alongside the proposed DRD-CN-DL model.

**Fig. 15:**
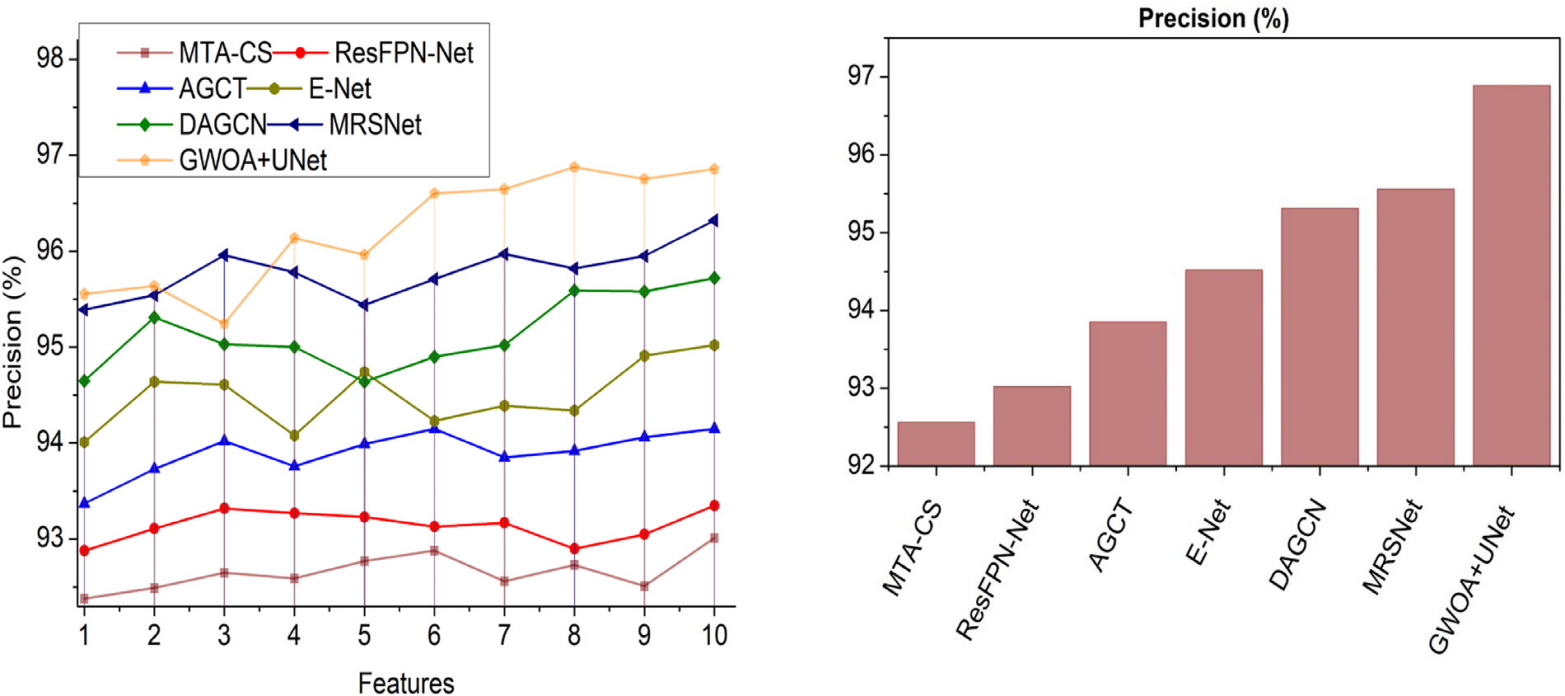
Precision.

**Fig. 16:**
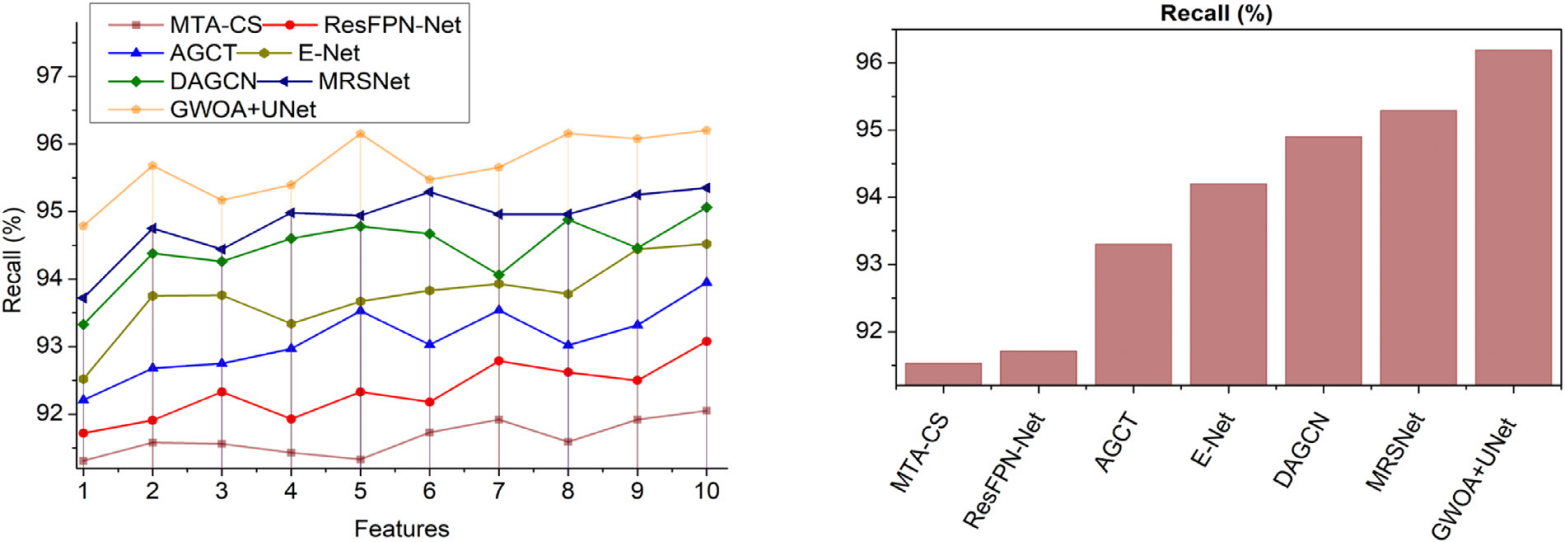
Recall.

**Fig. 17:**
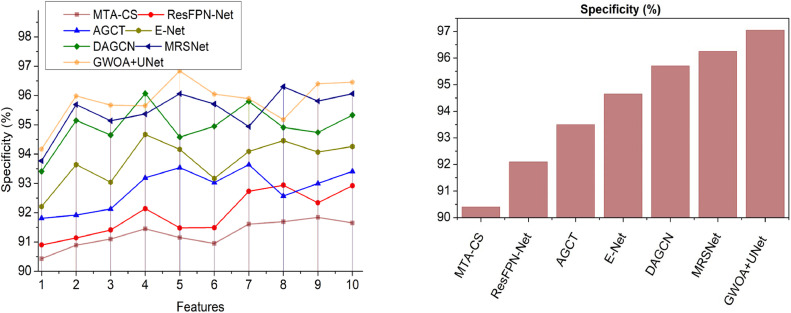
Specificity.

**Fig. 18:**
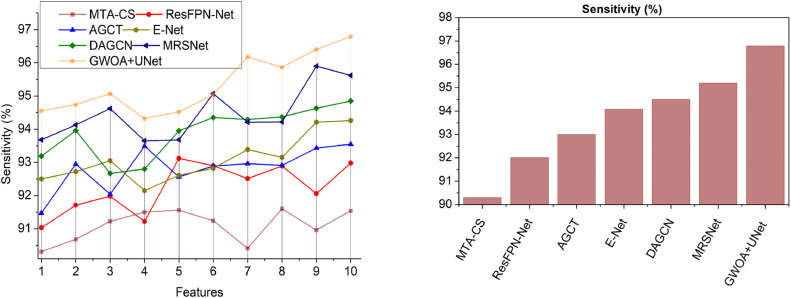
Sensitivity.

### Accuracy

In this proposal, the Grey Wolf Optimization is applied to improve the DR prediction and recognition accuracy at different time intervals (Refer to Fig. 14). The U-Net architecture is implemented to maximize the textural feature differentiation based on variation-causing and No variation features in original images and thereby reduces high differentiation identified features at the time of training. The sensitivity and specificity measure are balanced to achieve high-quality images with No variation-causing features is the optimal condition to improve accuracy. For instance, diabetic retinopathy prediction and recognition is performed using the GWO technique to identify diabetic mellitus on a retina and from which the DR is controlled. The proposed U-Net architecture relies on GWO for separately identifying encircling and pouncing based on its pooling layers for precise feature differentiation. This textural feature differentiation is used to control retina abnormalities using GWO. Similarly, the U-NET is implemented to identify variation-causing features based on dense convolution layers, upsampling, and downsampling for achieving high accuracy.

### Precision

In this model, the GWO utilized U-Net architecture is implemented to achieve high precision in DR detection. It is based on extracted textural features from the input retina images for precise diagnosis (Refer to Fig. 15). The variation-causing features are suppressed using U-Net architecture based on exploration and exploitation way. The feature differentiation is pursued to identify diabetic mellitus from the pre-processed images for improving diabetic retinopathy detection. Based on the maximum or minimum sensitivity and specificity features in the original images, the risk of eye structure variations is identified to improve retinal lesion segmentation. Accurate diabetic retinopathy prediction and recognition are performed to reduce the risk of vision loss. The U-Net architecture is implemented to maximize the semantic segmentation for precise diabetic retinopathy detection for increasing pooling layers during training. This model reduces the computational cost and time and thereby augments performance. In this scenario, the variation-identified features are recurrently trained until achieve maximum image segmentation. Hence, the high precision is achieved.

### Recall

In this DR prediction and recognition process is performed using a retina image for quality checking and textural feature differentiation to achieve high recall (Refer to Fig. 16). The Variation-causing features are addressed in the pre-processed image during the encircling process, and it is mitigated by using U-Net architecture. This U-Net performs the cross-connections from exploration to exploitation way to improve DR detection with high recall. The variation-identified features are extracted features for detecting the problems that impact the eye structure to improve accuracy and precision. The Grey Wolf Optimization helps to treat such eye structure variation issues with adaptable feature maps for achieving high-resolution images. For instance, accurate feature differentiation is pursued to identify the prominent metrics from original images; it reduces the chances of latency and feature mapping at the time of pack allocation. The U-Net architecture is implemented to achieve accurate retinal image quality assessment and abnormalities identification using fundus photography to increase segmentation. This GWO utilized U-Net architecture to achieve high sensitivity and specificity in DR detection. This reduces the problem occurrence and thereby augments recall.

### Specificity

In this contribution, sequential DR detection specificity is pursued based on textural feature extraction and differentiation as in Fig 17. In this architecture, the variations in each dimension are identified and mitigated by adding pooling layers. Based on textural feature differentiation, the GWO is applied to segment the images for retinal quality computation to achieve high specificity as compared to other factors. Using this model, the encoding, decoding, Downsampling, and Upsampling are computed to perform accurate DR prediction and recognition in the corresponding pre-processed images without increasing the variation occurred features is the optimal condition. The U-NET architecture is implemented to reduce the aforementioned problems in eye structure and thereby increase sensitivity and specificity. The architecture contains adaptable features that are mapping with an accurate network based on increasing the pooling layers levels. The extracted textural features from the original retina images are processed to provide an accurate diagnosis. Based on the optimization, high specificity is achieved.

### Sensitivity

The high-quality features in original images are identified using the GWO technique for improving DR prediction and recognition with high sensitivity (Refer to Fig 18). The pack allocation is pursued for reducing variation features in the pre-processed images. In this model, the DR is detected in specific region outputs in eye structure variation; such problems are identified through accurate image segmentation. A maximum pouncing with no variation is taken to achieve the high accuracy of retinal image detection. Routine training and updating are performed to address the textural variations in retina images with less time. Here, this validation is pursued to find the outcome range. In this quality computation, the random vectors between 0 and 1 range are used to improve image segmentation. The above sequence of feature differentiation is performed by using continuous training to prevent retina abnormalities. In this scenario, the input image is to be processed for addressing diabetic mellitus in the threshold regions to prevent variation occurrence. In this model, the dimension range is constantly defined to improve the accuracy from which the high sensitivity is achieved.

## Conclusion

This study introduces an advanced automated glaucoma screening system that integrates Grey Wolf Optimization Algorithm (GWOA)-enhanced U-Net++ for precise optic disc segmentation and Capsule Network (CapsNet) for robust glaucoma classification. By optimizing segmentation through GWOA's adaptive search mechanism and leveraging CapsNet's hierarchical feature extraction, our approach achieves superior diagnostic accuracy. With an impressive 96.01 % accuracy, the proposed framework outperforms conventional methods, demonstrating its potential for early glaucoma detection. This automated system not only enhances diagnostic efficiency but also addresses the limitations of manual screening, making it a scalable and practical solution for real-world clinical applications. By improving early detection, our method contributes to reducing the global burden of glaucoma-induced blindness, offering a transformative advancement in ophthalmic healthcare. Future work will focus on refining model generalizability across diverse datasets and integrating additional clinical parameters for enhanced diagnostic precision.

### Future work

(i) Enhancing Generalizability Across Diverse Datasets
  While our GWOA-UNet++ & CapsNet framework demonstrates high accuracy (96.01 %), further validation on larger, multi-ethnic, multi-source datasets is necessary to ensure robust generalization. Future research will explore domain adaptation and transfer learning techniques to improve model reliability across different imaging devices and population groups.
(ii) Integration of Multi-Modal Data for Comprehensive Diagnosis
  Current glaucoma detection relies primarily on fundus images but incorporating Optical Coherence Tomography (OCT) and visual field data could enhance diagnostic accuracy. Future work will explore multi-modal deep learning architectures that integrate structural and functional biomarkers for a more holistic assessment of glaucoma progression.
(iii) Real-Time Monitoring & Early Progression Prediction
  Developing a longitudinal glaucoma monitoring system using time-series deep learning models (e.g., LSTMs, transformers) could enable early-stage prediction of disease progression, facilitating proactive intervention before irreversible vision loss occurs.
(iv) Optimized Model Deployment for Clinical Use
  Ensuring low-latency, real-time analysis is critical for scalable telemedicine applications. Future work will focus on lightweight deep learning models optimized for mobile and edge devices, making glaucoma screening more accessible in remote and underserved areas.

While many AI models remain in research phases, our proposed method has strong potential for clinical implementation. By addressing these research gaps, our framework can evolve into a comprehensive, scalable, and clinically deployable solution for glaucoma diagnosis and monitoring.

## Limitations

• Dataset Constraints: While our model achieves 96.01 % accuracy, its performance has been validated primarily on fundus image datasets, limiting generalizability across different imaging modalities such as OCT or visual field tests.
• Computational Complexity: Though optimized, CapsNet requires higher computational resources than traditional CNNs, making deployment on low-power devices challenging.
• Limited Clinical Testing: The model has not yet been extensively tested in real-world clinical environments, requiring further validation on diverse patient populations.

## Ethics statements

In this Manuscript no, human participants or animals their data or biological material, are not involved.

## CRediT author statement

For the individual contribution of research author and co-authors as follows: **Govindharaj I:** Conceptualization, Methodology, Validation, Writing—original draft, Funding acquisition, Project administration. **Ramesh T:** Formal analysis, Writing—review & editing, Supervision. **Poongodai A:** Investigation, Resources. **Senthilkumar K.P:** Data curation, Formal analysis, Validation. **Udayasankaran P:** Visualization, Writing - original draft. **Ravichandran S:** Investigation, Software.

## Declaration of competing interest

The authors declare that they have no known competing financial interests or personal relationships that could have appeared to influence the work reported in this paper.

## Data Availability

No data was used for the research described in the article.
